# Supplementary material for: The Construction and Exploration of a Comprehensive MicroRNA Centered Regulatory Network in Foxtail Millet (Setaria italica L.)
Source: Front Plant Sci. 2022 May 6;13:848474. doi: 10.3389/fpls.2022.848474 (PMC9121102; doi:10.3389/fpls.2022.848474)
Supplement: Supplementary file 3 [file Data_Sheet_3.zip › Sit-miRN3002_Seita.1G126800.1_1781_TPlot.pdf]

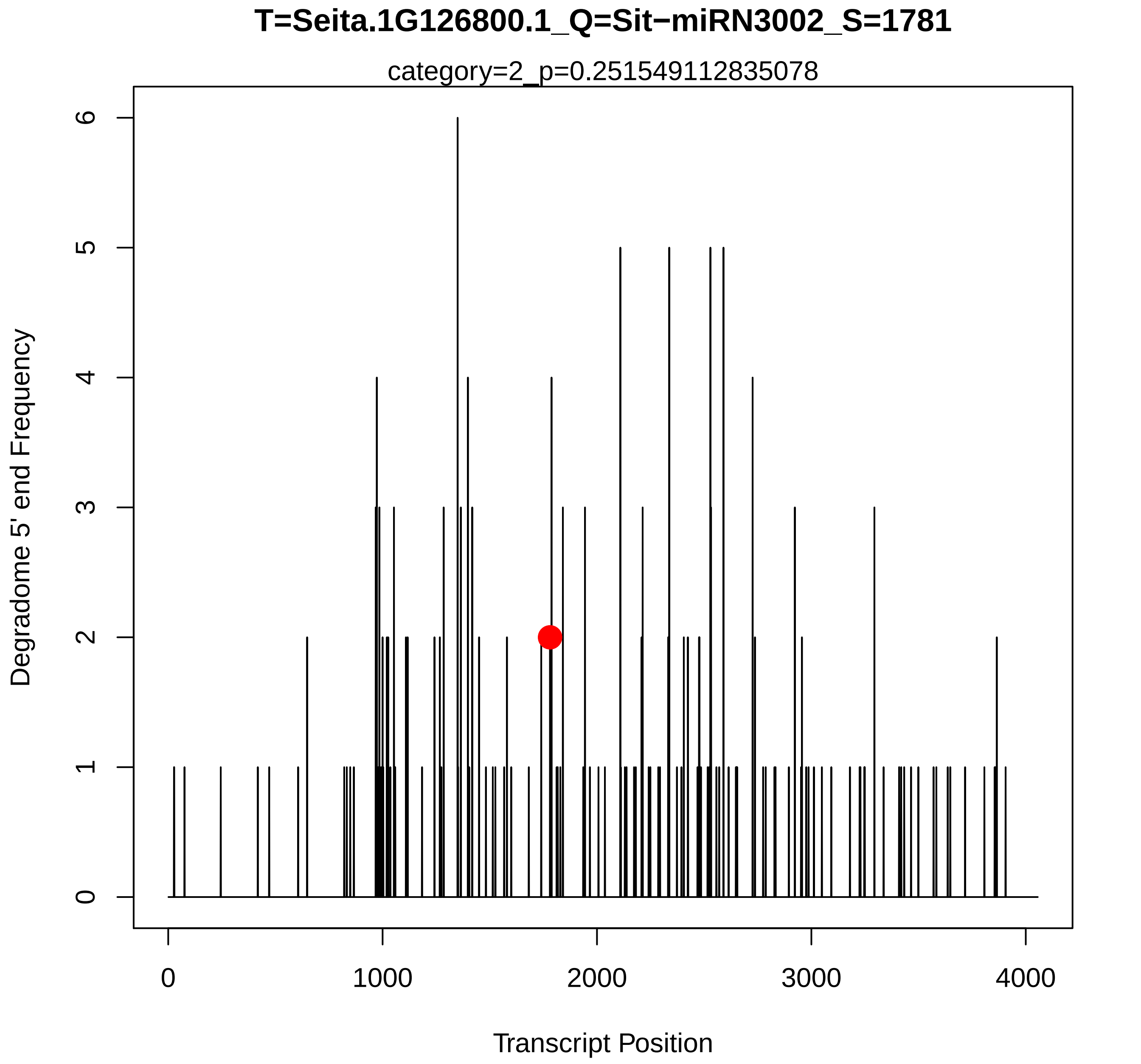
T=Seita.1G126800.1_Q=Sit−miRN3002_S=1781
category=2_p=0.251549112835078
Degradome 5' end Frequency
0
1
2
3
4
5
6
0
1000
2000
3000
4000
Transcript Position
